# Near-infrared exciton-polaritons in strongly coupled single-walled carbon nanotube microcavities

**DOI:** 10.1038/ncomms13078

**Published:** 2016-10-10

**Authors:** Arko Graf, Laura Tropf, Yuriy Zakharko, Jana Zaumseil, Malte C. Gather

**Affiliations:** 1 Institute for Physical Chemistry, Faculty of Chemistry and Earth Sciences, Universität Heidelberg, Im Neuenheimer Feld 253, D-69120 Heidelberg, Germany; 2 Organic Semiconductor Centre, SUPA, School of Physics and Astronomy, University of St Andrews, North Haugh, St Andrews KY16 9SS, UK

## Abstract

Exciton-polaritons form upon strong coupling between electronic excitations of a material and photonic states of a surrounding microcavity. In organic semiconductors the special nature of excited states leads to particularly strong coupling and facilitates condensation of exciton-polaritons at room temperature, which may lead to electrically pumped organic polariton lasers. However, charge carrier mobility and photo-stability in currently used materials is limited and exciton-polariton emission so far has been restricted to visible wavelengths. Here, we demonstrate strong light-matter coupling in the near infrared using single-walled carbon nanotubes (SWCNTs) in a polymer matrix and a planar metal-clad cavity. By exploiting the exceptional oscillator strength and sharp excitonic transition of (6,5) SWCNTs, we achieve large Rabi splitting (>110 meV), efficient polariton relaxation and narrow band emission (<15 meV). Given their high charge carrier mobility and excellent photostability, SWCNTs represent a promising new avenue towards practical exciton-polariton devices operating at telecommunication wavelengths.

## Abstract (web summary)

The formation of exciton-polaritons in organic semiconductors by strong coupling to a cavity is a promising route towards electrically pumped organic lasers. Here, Graf *et al*. demonstrate strong coupling from semiconducting single-walled carbon nanotubes in the near infrared frequency range.

## Graphical abstract

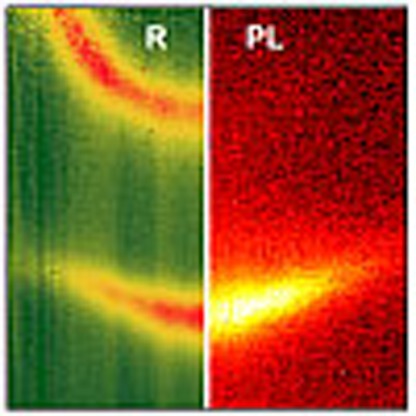

Exciton-polaritons are quasiparticles that have both light and matter character. They are formed when an optical microcavity is tuned to the excitonic absorption of an emitter within the cavity and the coupling between excited states of the emitter and cavity photons exceeds their rate of dissipation. In this strong coupling regime, the exciton and cavity dispersion show a characteristic anticrossing which gives rise to two new modes—the upper (UP) and the lower polariton (LP)^1^,^2^,^3^,^4^,^5^. Exciton-polaritons enable the study of exciting phenomena, such as Bose–Einstein condensation^6^, inversionless polariton lasing^7^,^8^,^9^, superfluidity^10^,^11^ and non-Hermitian topological structures^12^. The physics of polaritons has traditionally been studied using emitters and microcavities based on inorganic semiconductors. However, organic semiconductors, which are materials based on π-conjugated hydrocarbons, attract rapidly increasing attention in this context. The high oscillator strength of excitonic transitions in these materials can facilitate much larger Rabi splitting than in inorganic semiconductors^13^,^14^,^15^ and due to their large binding energy, the Frenkel-type excitons in organic materials offer a direct route to polariton condensation and polariton lasing at room temperature^16^,^17^,^18^. Electrically driven lasing has been a long standing, yet elusive goal in the organic semiconductor community^19^. The low thresholds of the recently reported organic polariton lasers have sparked hopes that electrical pumping may be feasible using exciton-polaritons. However, the organic materials investigated in the context of strong coupling may not provide sufficient charge carrier mobility. In addition, strong-coupling phenomena in organic materials have so far only been investigated in the visible part of the spectrum although devices operating in the near infrared (NIR) will be highly desirable for applications in telecommunication and for integration with silicon photonics.

Interestingly, semiconducting single-walled carbon nanotubes (SWCNTs) combine the advantageous excitonic properties of conventional organic semiconductors with extremely high-charge carrier mobilities^20^ (∼10,000 cm^2^ V^−1^ s^−1^) and emit in the NIR (ref. 21). SWCNTs show narrow linewidth emission (half width at half maximum, HWHM, in dispersion, ∼10 meV) (ref. 22), a large oscillator strength^23^ of ∼5 and their high exciton binding energies render excitons in SWCNTs stable at room-temperature^24^. The energetic position of the transition depends on the chirality vector (n,m), and can thus be easily adjusted across the entire NIR. Recently, these properties have been exploited in nanoscale devices based on individual SWCNTs, for example, for room-temperature single-photon sources^25^, integration into photonic waveguides^26^ and coupling to ultralow mode-volume photonic crystals^27^,^28^. On a microscopic scale, the luminescence of SWCNTs can be enhanced by orders of magnitude using photonic crystals^29^, and net optical gain has been reported for SWCNT waveguides^30^. However, substantial self-absorption of the strongly overlapping absorption and emission spectra (Stokes shift in a high-quality dispersion of SWCNTs^22^, ∼2 meV) limits emission efficiency in many situations and may complicate realization of efficient devices, for example, for conventional photonic lasing^23^. For polariton based devices, by contrast, the self-absorption of SWCNTs can be bypassed as the emission from the lower polariton branch in the strong coupling regime is expected to be red-shifted.

Here, we report strong exciton-photon coupling in metal-clad microcavities filled with purified monochiral (6,5) SWCNTs and show that Rabi splitting above 110 meV can be achieved at room temperature. Exciton-polaritons were observed by angle-resolved reflectance and polariton emission measurements and were found to agree well with the coupled oscillator model and transfer-matrix simulations. We also investigate the influence of the SWCNT concentration within the cavity on the strength of light-matter coupling and on the polariton relaxation efficiency. Our experiments highlight the potential of SWCNTs for studying exciton-polariton phenomena in the NIR and pave the way to future applications of polaritons, for example, in the telecom domain.

## Results

### SWCNT characterization

**Figure 1:**
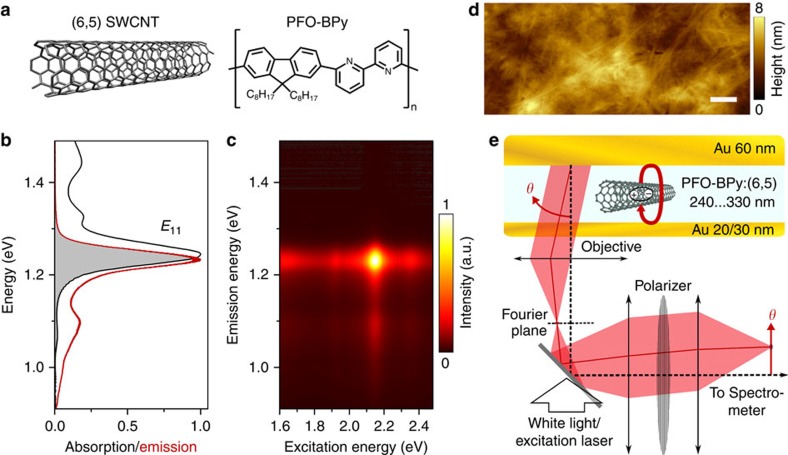
(6,5) Single-walled carbon nanotubes (SWCNTs) in thin polymer films. (**a**) Molecular structure of a (6,5) SWCNT and of the copolymer PFO-BPy. (**b**) Absorption and emission spectra of (6,5) SWCNTs embedded in a PFO-BPy polymer matrix. (**c**) PL excitation-emission map of a thin film of PFO-BPy embedded SWCNTs. (**d**) Representative topography of the film surface (tapping mode AFM; scale bar, 100 nm). (**e**) Setup used for strong coupling experiments containing the sample comprising SWCNT/PFO-BPy in a metal-clad cavity and Fourier imaging optics for angle resolved spectroscopy.

High purity (6,5) SWCNTs were enriched by selective wrapping with the copolymer PFO-BPy (Fig. 1a), and then embedded in a host layer of PFO-BPy at three different concentrations (0.20, 0.46 and 1.71 wt%; see Supplementary Table 1). To obtain large amounts of monochiral SWCNTs with high optical quality, sonication-free shear force mixing was used for dispersion (see Methods and ref. 22). Figure 1b shows the absorption and emission spectra of a thin film of PFO-BPy-embedded SWCNTs (1.71 wt%) that were fabricated in this way. The *E*_11_ absorption peak is located at 1.243 eV (997.5 nm) and is referred to as exciton (X) in the following. The *E*_11_ absorption and emission linewidths (HWHM of Lorentzian fit, 20.5 and 21.9 meV, respectively) are broadened compared to SWCNTs in dispersion (∼10 meV), but are still narrow compared to most organic materials. The Stokes shift is 8.7 meV, which is again smaller than for most organic semiconductors. SWCNT films have a background refractive index of around 1.65 and show a strong Lorentzian oscillation close to the *E*_11_ peak (Supplementary Fig. 1). Figure 1c shows a photoluminescence (PL) emission-excitation map of the film. Emission from chiralities other than (6,5) is negligible, confirming the high purity of the used SWCNTs. The strong *E*_22_ transition of the (6,5) SWCNTs at 2.149 eV (577 nm) facilitates efficient off-resonant excitation at visible wavelengths. We attribute the fact that the spectral characteristics and emission of our SWCNTs are well preserved in the film to excellent separation of neighbouring nanotubes by the wrapping polymer chains and by the polymer host. Atomic force microscopy (Fig. 1d for a representative topography map) confirms the high density of SWCNTs in our films and shows that films have a root-mean-square (RMS) roughness of ∼3 nm, which is sufficiently small for integration into microcavities with high optical quality.

### Strong light-matter coupling

Microcavities for strong coupling experiments were fabricated by depositing the SWCNT films (layer thickness, 240–330 nm) on a silicon wafer coated with a thick gold layer as a bottom mirror and depositing the second semi-transparent gold mirror on top (Fig. 1e and Methods). Light-matter coupling in these cavities was investigated via polarization and angle-resolved reflectivity and PL spectroscopy using the Fourier imaging system schematically illustrated in Fig. 1e.

**Figure 2:**
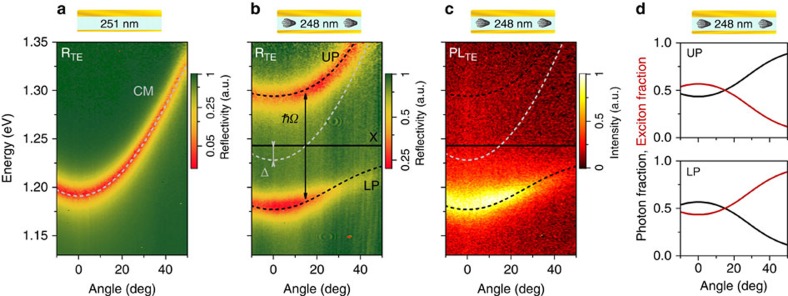
Strong coupling of SWCNTs in microcavities. (**a**) Angle and spectrally resolved reflectivity for a 251 nm thick reference cavity with the pure host polymer PFO-BPy. The grey dashed line indicates the fitted dispersion of the cavity mode for *n*_eff_=1.67 and *E*_0_=1.185 eV. (**b**) Angle- and spectrally resolved reflectivity of a 248 nm thick cavity containing PFO-BPy with embedded (6,5) SWCNTs. Strong coupling between *E*_11_ exciton (X, solid black line) and cavity photons (grey dashed line) leads to mode splitting into an UP (upper polariton) and LP (lower polariton) branch (black dashed lines, coupled oscillator model fits) with a characteristic Rabi splitting (*ħΩ*) of 113 meV. The detuning Δ between cavity mode and the exciton mode is −17 meV. (**c**) Angle- and spectrally resolved photoluminescence of the same cavity under 640 nm excitation showing exciton-polariton emission from the LP branch. (**d**) Exciton and photon fractions of UP (top) and LP (bottom) as a function of angle. All plots show the results for TE polarization (see Supplementary Fig. 2 for TM).

We first examined a reference cavity that contained only the host polymer PFO-BPy but no SWCNTs. In the TE polarized angle-dependent reflectivity spectrum of this structure, a cavity mode (CM) is clearly visible as a line of minimum reflectance (Fig. 2a; see Supplementary Fig. 2 for TM polarization). Excellent agreement between the experimentally observed position of the cavity mode and the expected cavity dispersion (see Methods, equation (2)) is obtained for a CM energy of *E*_0_=1.1850, eV and an effective refractive index of *n*_eff,TE_=1.67 (grey dashed line in Fig. 2a). For TM polarization, the best fit was achieved for a slightly higher effective refractive index (*n*_eff,TM_=1.95), in agreement with previous reports^4^. By comparing the experimental data to transfer-matrix simulations, we determined a cavity thickness of 251 nm (Supplementary Fig. 3a). The HWHM line width of the mode at 0° angle of incidence was 26 meV, yielding a quality factor of the cavity of *Q*=*E*/Δ*E*≈23.

Next, we investigated a cavity containing a composite film with a high density (1.71 wt%) of (6,5) SWCNTs. The cavity thickness was chosen such that the excitonic transition of the SWCNTs overlapped with the cavity mode. For this structure a splitting of the cavity mode into an UP and LP branch is observed, which is evidence for strong light-matter coupling in this system (Fig. 2b). The Rabi splitting (*ħΩ*) characterizing the coupling strength is given by the smallest energy difference between UP and LP. For the present example, the minimum UP–LP difference occurs at ±11° angle of incidence, and the corresponding Rabi splitting is 113 meV (Supplementary Fig. 3c). The angle-dependent reflection spectra of the SWCNT-filled cavity were fitted with the analytical solution of the UP and LP predicted by a simple coupled oscillator model^31^, using only *ħΩ* and the detuning (Δ) of the cavity with respect to the exciton as fitting parameters (see lines in Fig. 2b). For the example shown in Fig. 2b, we obtain *ħΩ*=116 meV, in good agreement with the directly measured value quoted above, and find that the cavity mode is detuned by Δ=−17 meV.

We also performed transfer-matrix simulations of the SWCNT-containing cavities to determine their thickness. For a 248 nm thick polymer-SWCNT layer, the predicted shape of the UP and LP agrees well with our experimental findings, with small deviations observed only at angles >30° (Supplementary Fig. 3b). In addition, the values for Rabi splitting and detuning agree with our earlier results (114 and −16 meV, respectively), corroborating the validity of the transfer-matrix algorithm and the refractive index data, as well as the optical quality of the SWCNT film.

The formation of exciton-polaritons requires that the light–matter interaction is faster than the decoherence time of the uncoupled oscillators themselves^31^. In the framework of our coupled oscillator model, strong coupling sets in once the Rabi splitting *ħΩ* is larger than the sum of the half width of the homogeneously broadened exciton mode *ħΓ*_X_ and the cavity mode *ħΓ*_C_ (see Methods)^1^,^31^. From earlier measurements of the fluorescence lifetime of SWCNTs in a polymer matrix^32^, we estimate the homogeneous broadening of the exciton to be *ħΓ*_X_≈0.4 meV, much smaller than the HWHM of the cavity mode (*ħΓ*_C_=26 meV). The minimum Rabi splitting required for strong coupling in our SWCNT microcavities is therefore *ħΩ*_min_=*ħΓ*_X_+*ħΓ*_C_≈27 meV. Although the simplifications used in the coupled oscillator model mean that in practice this condition not always distinguishes a sharp transition to strong coupling, the experimentally observed Rabi splitting of 113 meV clearly exceeds the above value and is thus clear evidence that the system is in the strong coupling regime. Note that in addition to this fundamental prerequisite, inhomogeneous broadening of the exciton mode will increase the minimum coupling required to observe anti-crossing of the involved modes^33^. When taking inhomogeneous broadening into account, the minimum splitting required to clearly resolve strong coupling is given by the sum of the spectral width of the two polariton branches, that is, *ħΩ*≥*ħΓ*_UP_+*ħΓ*_LP_=38 meV, which again is clearly fulfilled in our case. Depending on the signal-to-noise ratio even smaller splitting may be detectable by deconvolution.

To investigate the dynamics of the coupled system, we next investigated the angle-resolved PL spectrum of the cavity under off-resonance excitation with a 640 nm cw-laser (Fig. 2c). The PL follows the LP branch and no emission from the UP branch is observed. The peak wavelength of the PL emission from the hybrid state is significantly detuned relative to both the (6,5) SWCNT exciton and the cavity mode, confirming that the emission is from exciton-polaritons within the microcavity. We also calculated the photonic and excitonic fraction of the exciton-polaritons as a function of angle. As shown in Fig. 2d, the excitonic character increases with increasing angle for the LP branch and conversely decreases with angle for the UP branch.

**Figure 3:**
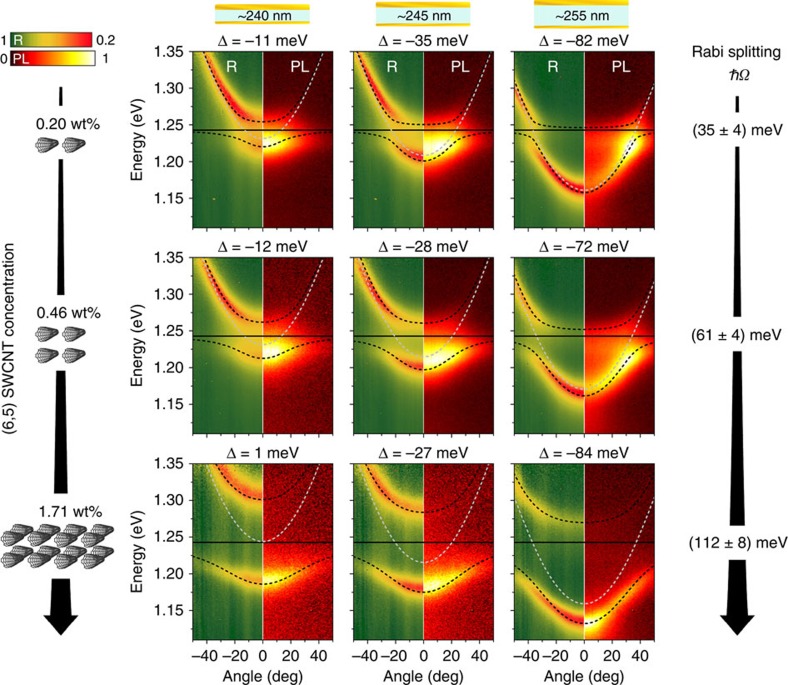
Concentration dependence of Rabi splitting in SWCNT microcavities. Angle- and spectrally resolved reflectivity (R) and photoluminescence (PL) for increasing concentrations of (6,5) SWCNTs (top to bottom) and increasing cavity thickness and detuning (left to right). Black solid lines indicate the *E*_11_ exciton of (6,5) SWCNTs. UP and LP (coupled oscillator model) are traced by dashed black lines and the simulated cavity mode by a grey dashed line. On the right, the Rabi splitting obtained by averaging over more than ten cavity thicknesses is shown for each concentration. All plots show the results for TE polarization (see Supplementary Fig. 4 for TM).

To fully explore the properties of SWCNTs in the strong coupling regime, we characterized cavities with three different SWCNT concentrations and a range of different thicknesses, that is, with slightly different detunings Δ. Figure 3 summarizes the measured angle- and spectrally resolved reflectivity and PL for the three concentrations (top to bottom) and for three cavity thicknesses (left to right). While a clear anti-crossing of the cavity mode and the exciton is observed in all measurements, the magnitude of the splitting increases significantly with SWCNT concentration. To obtain a robust estimate of the Rabi splitting achievable for each SWCNT concentration, the splitting was averaged across more than ten different cavity thicknesses. The average Rabi splittings extracted from the measurements were 35±4 meV, 61±4 meV and 112±8 meV for SWCNT concentrations of 0.20 wt%, 0.46 wt% and 1.71 wt%, respectively. (The uncertainties in this measurement result from small inhomogeneities of local SWCNT concentrations and from the thickness-dependence of the Rabi splitting.)

For all SWCNT concentrations investigated here, the observed Rabi splitting is larger than *ħΩ*_min_ and thus all samples are in the strong coupling regime. However, the angle-resolved emission spectra vary significantly for different concentrations and detunings. For the lowest SWCNT concentration (0.20 wt%), the observed cavity emission deviates significantly from the LP branch observed in reflection. In case of negative detuning (Δ=−82 meV), most polaritons do not relax at *k*=0 but instead emit at higher angles of ∼35°. This is evidence of a polariton relaxation bottleneck similar to the observations made in other organic materials^3^. Furthermore, at the bottleneck position the emission spectrum is red-shifted by 24 meV compared with the free space emission of the SWCNTs and the polaritons have low excitonic fraction (Supplementary Fig. 5), which is evidence against a purely molecular origin of the bottleneck. Increasing the SWCNT concentration to 0.46 wt% increases the Rabi splitting to 61 meV and reduces the observed bottleneck effect. For the highest concentration investigated here (1.71 wt%), the Rabi splitting is boosted to 112 meV and no bottleneck is observed anymore. We attribute the increased relaxation efficiency to the larger excitonic character of the exciton-polaritons in the LP at small angles (Supplementary Fig. 5) as discussed by Coles *et al*.^3^. A larger excitonic fraction improves relaxation due to increased polariton lifetime and the resulting flatter dispersion supports polariton-phonon scattering^34^.

## Discussion

**Figure 4:**
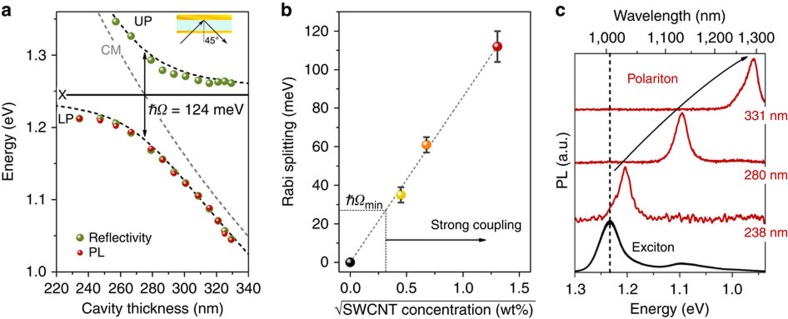
Concentration dependence of strong coupling and polariton tuning. (**a**) Reflectivity and PL of SWCNT-filled cavity at 45° angle of incidence as a function of cavity thickness. Coloured spheres are experimentally determined values for UP and LP from the angular reflectivity/PL measurements at different cavity thicknesses (raw data partially shown in Fig. 3). The black dashed line shows the transfer matrix simulation. The grey dashed line is the cavity mode. (**b**) Experimentally determined Rabi splitting versus square root of SWCNT concentration and linear fit to data. The error bars indicate the standard deviation of the mean (Fig. 3). (**c**) Polariton emission spectra in forward direction for three different cavity thicknesses (red) in comparison to the uncoupled emission spectrum (black). All plots are for TE polarization.

From the measurements presented here, we can extract the thickness-dependent reflectivity of the cavity for any angle of incidence (within ±0.8° resolution given by the finite size of the spectrometer slit). As an example, Fig. 4a shows the position of the UP (reflectivity) and LP (reflectivity and PL) *versus* thickness at 45° angle of incidence. The experimental data agree well with the prediction of transfer-matrix simulations (shown as dashed lines). The fit to the data gives a Rabi splitting of 124 meV, which is slightly higher than the values found above for smaller angle of incidence and the same SWCNT concentration (112 meV). This discrepancy is due to the increased effective cavity length at 45° angle of incidence. (The effective cavity length is given by *d*_0_/sin(45°) with *d*_0_ being the actual thickness of the SWCNT layer.) This increase results in a larger number of SWCNTs coupling to the cavity and—because the Rabi splitting is proportional to the square root of the number of oscillators—to an increased Rabi splitting.

Rabi splitting generally scales with the square root of the oscillator strength in the cavity^13^,^35^,^36^ and so by using a large number of oscillators, the Rabi splitting could be increased further. Figure 4b summarizes the experimentally observed Rabi splitting as a function of the square root of the SWCNT concentration, showing a clear linear trend. From this plot, we estimate that for our cavity configuration a minimum SWCNT content of 0.11 wt% is required to obtain sufficient Rabi splitting to enter the strong coupling regime (that is, *ħΩ*>*ħΩ*_min_=27 meV). Furthermore, by extrapolating the data to higher concentrations, we predict that for 100 wt% SWCNTs a Rabi splitting of ∼850 meV should be achievable. This corresponds to ∼70% of the energy of the *E*_11_ transition and thus would put SWCNTs well in the ultrastrong coupling regime^4^. Under these conditions, we expect the polariton-emission of the cavity to be almost perfectly dispersionless. Cavities with angle-independent absorption and emission may offer exciting opportunities for SWCNT-based devices.

Applications in optical communications require emission to be within certain spectral bands (for example, O-band ∼1,300 nm or C-Band ∼1,550 nm). Using microcavities based on monochiral SWCNTs in the strong coupling regime facilitates wavelength tuning of the emission across a significant part of the NIR. Figure 4c shows the polariton-emission spectra in forward direction (angle of incidence, *θ*=0°) for three different cavity thicknesses (1.71 wt% (6,5) SWCNT concentration). Narrow emission peaks (<15 meV HWHM) are detected from 1,030 nm up to 1,300 nm, and there is no emission from the uncoupled exciton, which would occur at 1,010 nm, indicating that all nanotubes contribute to the strong-coupling phenomenon. Therefore, even with just the (6,5) chirality of SWCNTs, a spectral range of >300 meV can be covered. Adding a small number of other chiralities would be sufficient to cover the entire telecommunication band with SWCNT-based polariton emission.

In conclusion, our study opens up new avenues for employing the unique properties of SWCNTs and it demonstrates exciton-polaritons in the NIR at room temperature and for a carbon-based material. This is a direct consequence of the large oscillator strength of SWCNTs and of their well-defined and narrow exciton mode. The results obtained so far indicate that SWCNTs also show potential for realizing ultrastrong coupling. By selecting SWCNTs with different chiralities, the polariton energy could be tuned across the entire optical telecommunication band. In addition, unlike most organic semiconductors, SWCNTs are largely immune to photobleaching effects, rendering them particularly attractive for work requiring excessive exciton densities. Finally, the high charge carrier mobility of SWCNTs, which has been previously exploited to realize high-performance and light-emitting field-effect transistors^37^,^38^, is likely to enable electrically driven polariton devices in the NIR.

## Methods

### SWCNT enrichment and cavity structure

Dispersions of (6,5) SWCNTs in toluene were prepared from CoMoCAT raw material (773735, Lot #14J017A1, Sigma-Aldrich) as described in ref. 22. Briefly, 0.5 g l^−1^ PFO-BPy (poly((9, 9-dioctylfluorenyl-2, 7-diyl)-*alt-co*-(6, 6′-{2, 2′-bipyridine})), American Dye Source, *M*_W_=34 kg mol^−1^) was dissolved in 140 ml toluene before adding 0.38 g l^−1^ CoMoCAT. Shear force mixing (Silverson L5M-A) was then applied at maximum speed (10,230 r.p.m.) for 48 h. During mixing the temperature was kept constant at 20 °C. The dispersion step was followed by centrifugation at 60,000*g* (Beckman Coulter Avanti J26XP centrifuge) for 45 min with an intermediate supernatant extraction and centrifuge tube exchange after 15 min. The final supernatant contains high purity (6,5) SWCNTs that were further enriched by pelleting the dispersions via ultracentrifugation at 284,600*g*. To achieve the desired film thicknesses, the pellets were redispersed in PFO-BPy (25 g l^−1^ in toluene) using mild bath sonication for 5 min. SWCNT concentration was adjusted by dispersing different amounts of pellets. The final dispersion had an *E*_11_ absorbance of up to 240 cm^−1^ corresponding to 0.43 g l^−1^ of (6,5) SWCNTs (Supplementary Table 1).

A 60 nm thick Au mirror was thermally evaporated (Univex 350G) onto a polished Si wafer covered with 2 nm Cr for adhesion. Thin polymer films containing nanotubes were spin-coated onto this mirror at moderate speeds, between 800 and 1,500 r.p.m. The cavity was finalized by evaporating a Au mirror on top (with the thickness optimized for contrast of the formed modes). For the PFO-BPy reference and for samples with 1.71 wt% SWCNTs the top mirror had a thickness of 30 nm and for all other cavities the top mirror thickness was 20 nm. Due to the low spin speeds, thickness gradients across the samples were formed. Using these long-range thickness variations of the cavity architecture, allowed us to tailor the detuning by moving the spot under investigation (diameter, ∼2 μm) across the sample. This meant that it was possible to perform all measurements for a given concentration on the same sample which in turn minimized concentration inhomogeneities. (Thickness variations of the cavity across the ∼2 μm spot diameter were negligible.)

### Fourier imaging

For angle-resolved reflectivity measurements, the emission of a calibrated lamp was focused onto the sample by an NIR corrected × 100 objective with 0.8 NA (Olympus LMPL100xIR). The resulting spot diameter of ∼2 μm defines the investigated area on the sample. For PL measurements, the white light source was replaced by a laser diode (OBIS, Coherent Inc., 640 nm, cw, ∼10 mW). The light reflected/emitted by the cavity was imaged onto the entrance slit of a spectrometer (Princeton Instruments IsoPlane) using a 4*f* Fourier imaging system and was recorded by a 640 × 512 InGaAs camera (Princeton Instruments, NIRvana:640ST). Additionally, a long-pass filter (cut-off wavelength, 850 nm) and a linear polarizer were placed in front of the spectrometer. Angle calibration of the system was done by fitting the measured cavity mode of a reference sample of known refractive index.

### Simulation

To model the experimentally obtained angle-resolved reflectivity, we applied a simple coupled oscillator model^31^. The coupling between exciton 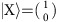 and photon 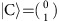 in a microcavity can be modelled by the coupled oscillator model with the Hamiltonian given by^31^


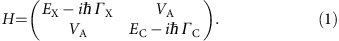


*E*_X_ is the energy of the *E*_11_ excitonic transition having a homogenously broadened HWHM *ħΓ*_X_. The energy dispersion of the cavity is given by


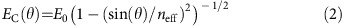


for a cavity tuned to *E*_0_=*E*_X_+Δ with Δ being the cavity detuning and *ħΓ*_C_ the HWHM of the cavity mode. The effective refractive index *n*_eff_ was set to the refractive index of the polymer host material (1.67 for TE and 1.955 for TM polarization). The coupling potential *V*_A_ of the two oscillators is related to the Rabi splitting at *E*_C_=*E*_X_ by 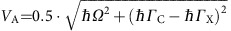.

The eigenvalues of the Hamiltonian


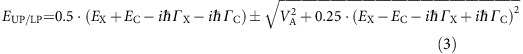


represent the UP and LP branch with the eigenvectors 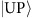 and 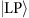. The photonic (excitonic) fractions *α*(*β*) of the new eigenstates can be calculated by their projection onto the old exciton and cavity modes, that is, 
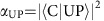
 and 
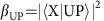
 for UP and analogous for LP. Following ref. 31, the strong coupling regime is reached when 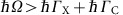, which is equal to 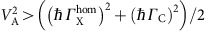 (ref. 1).

Transfer-matrix simulations were performed, based on complex refractive index data obtained by spectroscopic ellipsometry measurements of the pure host polymer and of a composite film containing 1.71 wt% of (6,5) SWCNTs. To allow for parametrization of the SWCNT concentration in the refractive index data, the contribution of SWCNTs to the overall refractive index was approximated by a Lorentzian absorption spectrum using Kramers–Kronig relations (Supplementary Fig. 1). In the corresponding fitting routine, based on transfer matrix simulations, the cavity thickness and the SWCNT concentration (Supplementary Table 1) were fitted.

### Data availability

All data is available at http://dx.doi.org/10.17630/2baaf9f7-9e91-456a-ae0c-22f752d7f7b6.

## Additional information

**How to cite this article:** Graf, A. *et al*. Near-infrared exciton-polaritons in strongly coupled single-walled carbon nanotube microcavities. *Nat. Commun.* **7,** 13078 doi: 10.1038/ncomms13078 (2016).

## Supplementary Material

Supplementary Information Supplementary Figures 1-5 and Supplementary Table 1
