# Supplementary material for: Near-infrared exciton-polaritons in strongly coupled single-walled carbon nanotube microcavities
Source: Nat Commun. 2016 Oct 10;7:13078. doi: 10.1038/ncomms13078 (PMC5062498; doi:10.1038/ncomms13078)
Supplement: Supplementary Information — Supplementary Figures 1-5 and Supplementary Table 1 [file ncomms13078-s1.pdf]

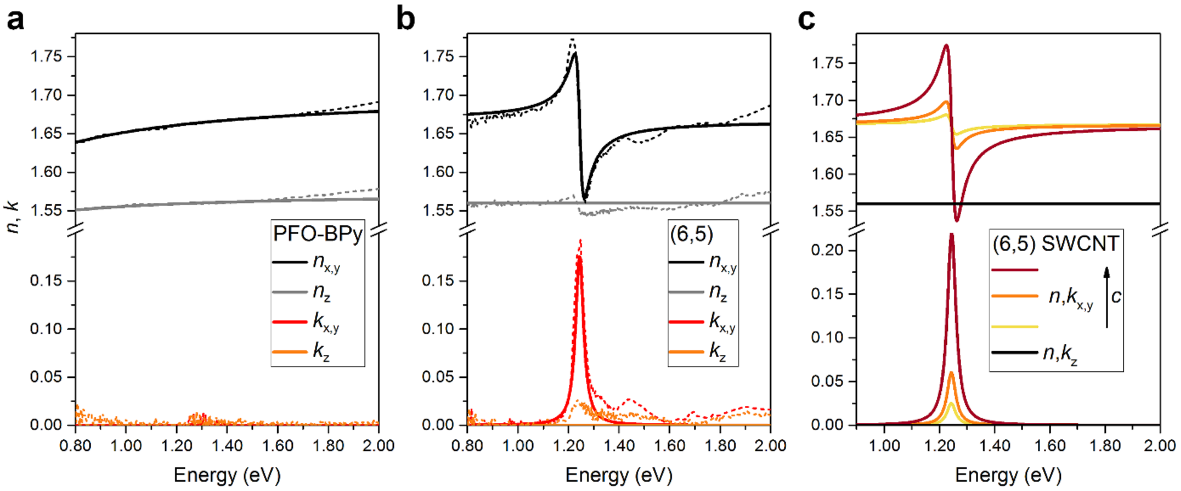

Supplementary Figure 1 | Complex refractive index. Real and imaginary part of the refractive index of (**a**) PFO-BPy and (**b**) (6,5) SWCNTs embedded in PFO-BPy. Data sets used for simulations (solid lines) and non-smoothed ellipsometry data (dashed lines). PFO-BPy and SWCNTs both show an anisotropic refractive index. For SWCNTs the *z*-component, here out-of plane, is neglected and thus perfect horizontal orientation of the tubes assumed. (**c**) The refractive index of the polymer layer containing different concentrations of (6,5) SWCNTs was approximated by modelling the imaginary part with a Lorentzian peak at the transition energy (peak width derived from ellipsometry data in b). The real part of the refractive index was deduced from the imaginary part through the Kramers-Kronig relation, using a dispersionless background refractive index of 1.67.

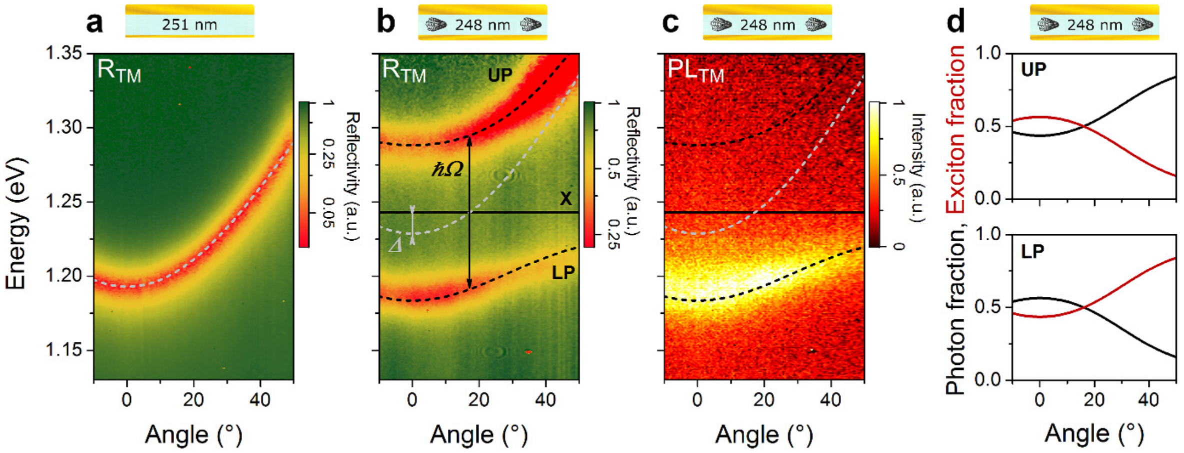

Supplementary Figure 2 | Strong coupling of SWCNTs in microcavities. (**a**) Angle- and spectrally resolved reflectivity for a 251 nm thick reference cavity with the pure host polymer PFO-BPy. The grey dashed line indicates the fitted dispersion of the cavity mode for $n_{\mathrm{eff}} = 1.955$ and $E_0 = 1.1844$ eV. (**b**) Angle- and spectrally resolved reflectivity of a 248 nm thick cavity containing PFO-BPy embedded (6,5) SWCNTs. Strong coupling between E_{11} exciton (X, solid black line) and cavity photons (grey dashed line) leads to mode splitting into an UP and LP branch (black dashed lines, coupled oscillator model fits) with a characteristic Rabi splitting ($\hbar\Omega$) of 102 meV. The detuning Δ between cavity mode and the exciton mode is −17 meV. (**c**) Angle- and spectrally resolved photoluminescence of the same cavity under 640 nm excitation showing exciton-polariton emission from the LP branch. (**d**) Exciton and photon fraction of UP (top) and LP (bottom) as function of angle. All plots show the results for TM.

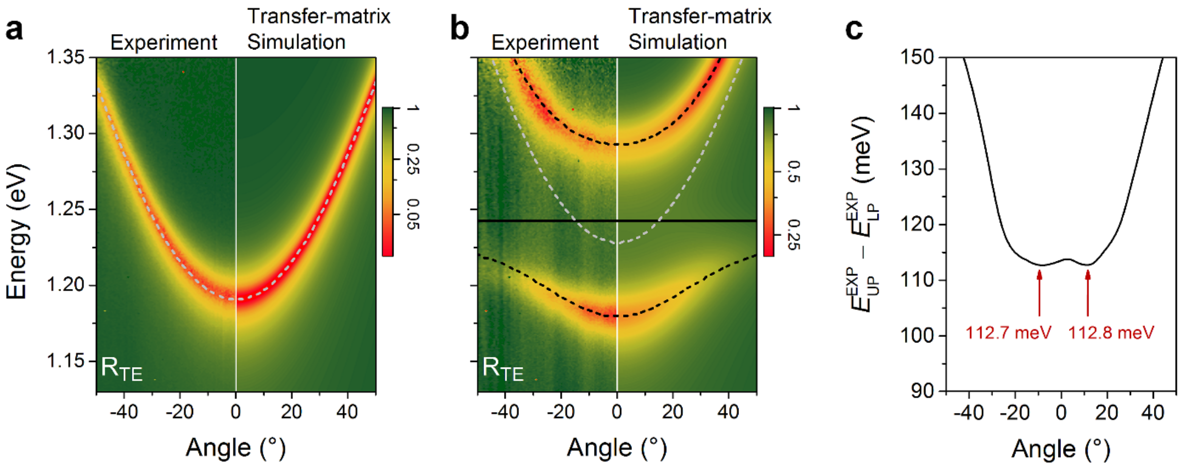

Supplementary Figure 3 | Transfer-matrix simulations and Rabi splitting. (**a**) Comparison of measured (left) and simulated (right, transfer-matrix algorithm) angular TE-polarized reflectivity of a reference cavity containing only PFO-BPy. (**b**) Same comparison for a cavity containing (6,5) SWCNTs. (**c**) Example illustrating how the Rabi splitting is determined by subtracting the LP mode from the UP mode, leading (for negative detuning) to two minima, as indicated by the red arrows.

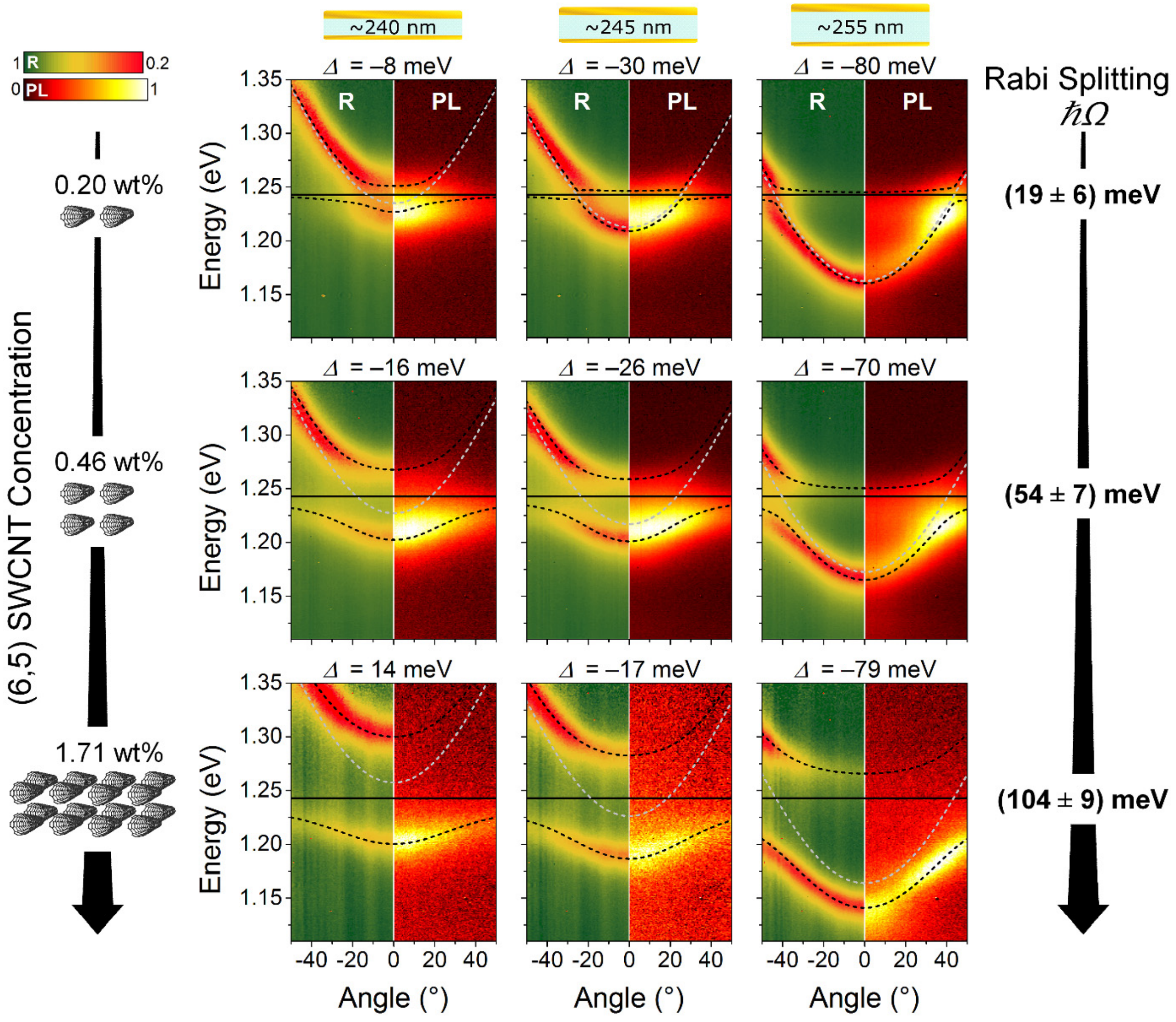

Supplementary Figure 4 | Concentration dependence of Rabi splitting in SWCNT microcavities (only TM polarization). Angle and spectrally resolved reflectivity (R) and photoluminescence (PL) for increasing concentration of (6,5) SWCNTs from top to bottom and increasing cavity thickness from left to right. Black solid lines indicates the E_{11} exciton of (6,5) SWCNTs. UP and LP (coupled oscillator model) are traced by dashed black lines and the simulated cavity mode by a grey dashed line. On the right, the Rabi splitting obtained by averaging over more than ten cavity thicknesses is shown for each concentration. All plots show the results for TM polarization.

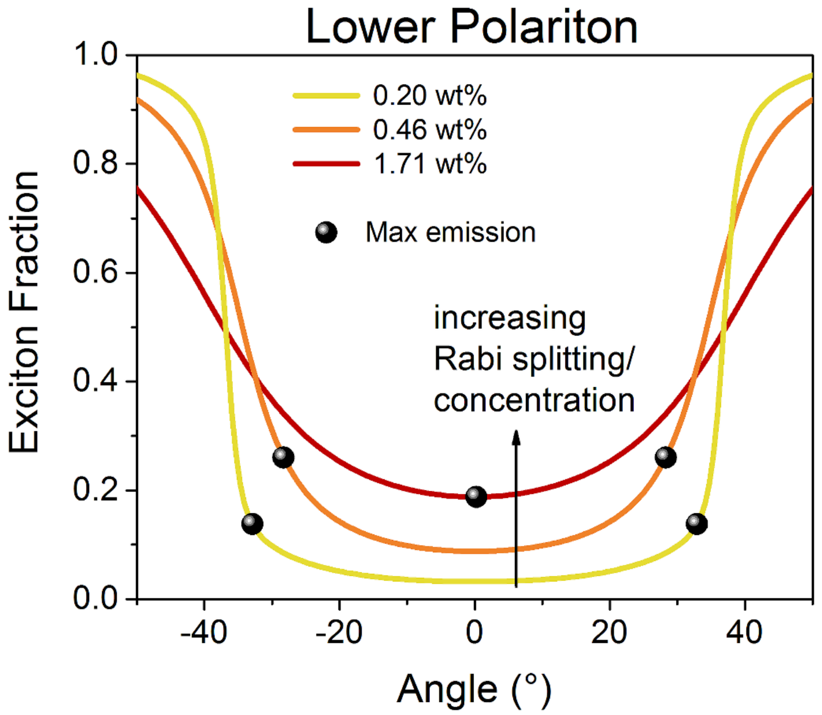

Supplementary Figure 5 | Concentration dependence of exciton fraction. Simulated exciton fraction of the lower polariton for negatively detuned cavities ($\Delta \approx -80$ meV shown in main text Fig. 3, right column). The increased coupling strength leads to higher excitonic content of the LP for small angles which favours efficient relaxation. The black spheres indicate the angle under which maximum polariton emission was observed.

Supplementary Table 1 | Summary of the (6,5) SWCNT concentrations used in this work.

Sample	Direct absorption measurement (in solution)			Transfer matrix simulation based fit (in thin film)		
	Abs @ E_{11}		$c_{(6,5)}$ / $c_{\text{PFO-BPy}}$	Average k_{max} (10 thicknesses)		Abs @ E_{11}
	(cm^{-1})	Relative	(wt%)		Relative	(μm^{-1})
**1**	28.1	**1**	0.202	0.0251	**1**	0.133
**2**	63.6	**2.6**	0.458	0.0603	**2.4**	0.324
**3**	236.8	**8.4**	1.705	0.2202	**8.8**	1.172
